# Zone-specific reference ranges of fetal adrenal artery Doppler indices: a longitudinal study

**DOI:** 10.1186/s12884-020-03480-z

**Published:** 2020-12-11

**Authors:** Ran Xu, Ziling Zhu, Wenjuan Tang, Qichang Zhou, Shi Zeng

**Affiliations:** 1 Department of Urology, The Second Xiangya Hospital, Central South University, Changsha, 410011 Hunan China; 2 Department of Ultrasound Diagnosis, The Second Xiangya Hospital, Central South University, 139 Renmin Road (M), Changsha, 410011 China

**Keywords:** Adrenal artery, Fetal, Pulsatility index, Doppler

## Abstract

**Background:**

The fetal adrenal gland is a highly vascularized organs and develops two recognizable distinct zones in uetro, inner fetal zone (FZ) and outer definitive zone (DZ). Based on the region supplied, middle adrenal artery (MAA) mainly contribute to FZ while inferior adrenal artery (IAA) mainly to the inferior part of DZ. The purpose of this study was to establish reference ranges of adrenal artery Doppler indices of IAA and MAA, and assess zonal difference of blood supply to fetal adrenal gland.

**Methods:**

The pulsatility index (PI), resistance index (RI), and systolic:diastolic ratio (S/D) of the IAA and MAA were obtained serially at 4-week intervals in normal fetuses. The MAA and IAA were referred based on the course and location in the gland: IAA referring the artery that mainly branches from the renal artery and walks along the renal upper pole, distributing the inferoposterior part of DZ in the adrenal gland while MAA as arterial blood flowing along the single central adrenal vein in the medial part of the gland. Multilevel modeling was performed to establish the gestational age-associated reference ranges for IAA and MAA. Differences in Doppler indices between the IAA and MAA were assessed.

**Results:**

One hundred sixty-eight fetuses with 843 observations were included. The IAA had a higher detection rate than the MAA (100% vs 89.2%, *p* < 0.05). The resistance of IAA had a reduction around 35 weeks of gestation and that of MAA remained unchanged throughout the second half of pregnancy. Lower PI, RI and S/D were observed in the MAA than in the IAA (*p* < 0.05) from 752 paired measurements.

**Conclusion:**

There is a zonal difference in blood supply in favor of the fetal zone, which may correspond to its unique function. Reference ranges of Doppler parameters in adrenal artery maybe beneficial for further evaluation of fetal hemodynamics.

## Background

The adrenal gland is one of the most highly vascularized organs in the human fetus [1]. The extensive vasculature in the fetal adrenal gland is crucial for gland cortex growth and steroidogenesis. The human adrenal gland develops two distinct zones by 50–52 days post conception [1, 2]: the inner fetal zone (FZ), occupying a large portion of the inner cortex, and the outer definitive zone (DZ), a narrow band surrounding the fetal zone. These two zones maintain entirely different morphologies and steroidogenesis patterns during the fetal period [1]. The FZ, a unique feature of fetal adrenals, is believed to be the site of abundant ∆5-steroid production and develops the zona reticularis. The DZ has been suggested as the site of aldosterone synthesis and likely forms the zona glomerulosa. In addition, the transitional zone (TZ), a very thin cortical zone between the FZ and DZ, has been demonstrated by ultrastructural studies [3] from the second trimester and has been suggested to be analogous to the zona fasciculate [2].

Routine obstetric ultrasound can detect the fetal adrenal gland from the second trimester. The FZ is identified as a hyperechogenic area in the center of the gland [4]. The DZ and TZ, together named the neocortex [5], are identified as the outer hypoechogenic areas surrounding the FZ. Although the blood flow of the fetal adrenal gland has been investigated previously by Doppler ultrasound [6–9], all of those investigations focused only on the middle adrenal artery (MAA) and were cross-sectional studies. The arterial vessels were classified as the superior, middle and inferior adrenal arteries based on their supply regions of the adrenal gland [10]. Briefly, the superior adrenal artery (SAA) reached the superior part of the gland, which is the location of the superior outer zone; the MAA reached the middle part of the gland in the area of the FZ; and the inferior adrenal artery (IAA) reached the inferior part of the gland, which is the location of the inferior outer zone. According to their course and small branches to the region on autopsy examination [10], we could speculate MAA mainly contribute to FZ while SAA and IAA mainly to the superior and inferior part of DZ. There was no investigation of the blood flow supply characteristics in the different adrenal zones.

Therefore, the purpose of this study was to establish reference ranges of Doppler indices (pulsatility index (PI), resistance index (RI), and systolic:diastolic ratio (S/D)) using serial measurement of adrenal arteries in normal fetuses, and to assess zonal and sex differences in blood supply to the fetal adrenal gland.

## Methods

A prospective longitudinal study was conducted at the Second Xiangya Hospital of Central South University in China between January 2019 and November 2019. Data on normal fetuses were collected from low-risk and healthy pregnant women. The gestational age (GA) was estimated from the day of the last menstrual period and was confirmed by an ultrasound measurement of the crown–rump length during the first trimester. The inclusion criteria were as follows: singleton pregnancies; GA from 20 to 24 weeks; absence of identified structural and chromosomal defects, absence of oligohydramnios or polyhydramnios, absence of fetal growth restriction and absence of maternal metabolic and systemic disease. All these fetuses were examined serially at 4-week intervals between 20 and 42 gestational weeks. Sex was identified after delivery. Written informed consent was obtained from all families, and this study was approved by the institutional review board of the Second Xiangya Hospital of Central South University (2018-Yan107).

Routine obstetrical ultrasound scans were performed by one investigator (ZJW) using a Voluson E10 (GE Healthcare Ultrasound, Milwaukee, WI, USA) ultrasound system with an RAB 4–8-D probe. Fetal biometry parameters, such as biparietal diameter, head circumference, femur length and abdominal circumference, were measured and used to calculate the estimated fetal weight (EFW). UA-PI and MCA-PI were obtained with insonation angle below 10 degree. Standard multiple views of the fetal heart were acquired by one expert (ZQC) to evaluate the cardiac anatomy.

**Fig. 1:**
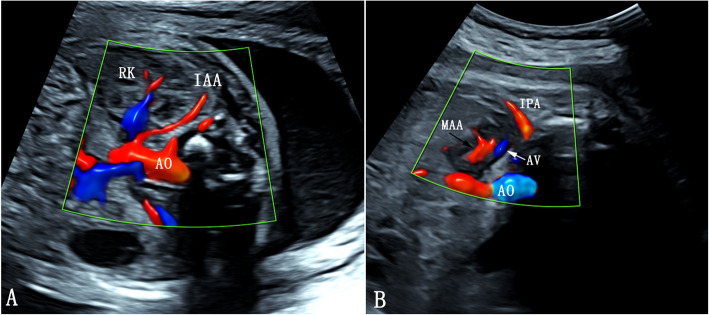
The color Doppler of A) the inferior adrenal artery and B) the middle adrenal artery. **a** IAA mainly branches obliquely from the proximal segment of the renal artery and then travels along the upper pole of the kidney with a curved course, supplying the posterior and inferolateral outer zone of the gland. **b** MAA travels along the single central adrenal vein in the medial part of the gland. IAA, inferior adrenal artery; RK, right kidney; AO, aorta; MAA, middle adrenal artery; AV, adrenal vein; IPA, inferior phrenic artery

The adrenal gland ultrasound was performed by one operator (ZS) who was blinded to the clinical data. Only the blood flow of the adrenal gland located in the ultrasonic near field was measured and recorded, regardless of whether it was the left or right gland. This study was designed to observe the Doppler indices of the MAA and IAA, not including that of the SAA, because the SAA is a very small branch mainly arising from the inferior phrenic artery [10] and cannot be detected by prenatal ultrasound. The MAA and IAA were identified according to their course and supply region in the gland but not their origin [10]. The MAA was identified as arterial blood flowing along the single central adrenal vein in the medial part of the gland on an oblique coronal view. The IAA was referred as the artery branching from the renal artery, walking along the renal upper pole and distributing the inferoposterior part of DZ in the oblique cross plane (Fig. 1). The insonation angle was kept among 0–10°. In the absence of fetal movements and maternal breathing, three to five uniform flow velocity waveforms were obtained for auto trace measurement. The following Doppler parameters were obtained: pulsatility index (PI), resistance index (RI), and systolic:diastolic ratio (S/D). Doppler indices of both MAA and IAA were measured three times, and the mean was used for the analyses.

The data were analyzed using STATA 15 (Stata Corp LLC, College Station, TX). The Shapiro-Wilk W test was performed to assess the normality of the distribution. Box Cox transformation was performed for PI, RI and S/D to calculate the appropriate lambda (λ) to reduce heteroscedasticity of residuals and non-parallelism [11]. Fractional polynomials were performed to find the best-fitting curves between adrenal gland Doppler indices and GA. Multilevel modeling with the restricted maximum likelihood option was used to estimate GA-specific reference percentiles [11]. Comparison between the MAA and IAA was performed using a paired samples t test. The Doppler indices were compared among gestational weeks using one-way analysis of variance (ANOVA) with post hoc Games-Howell testing. Comparison of the Doppler indices between male and female fetuses was performed for each gestational week using an independent samples t test. The intraclass correlation coefficient (ICC) was used to assess the interobserver agreement for 80 randomly selected observations. *P* < 0.05 was considered statistically significant.

## Results

Measurement of fetal adrenal arteries was attempted in 178 singleton pregnancies. Ten cases were excluded because of 4 cases with missing follow-up, 4 cases with maternal pre-eclampsia and gestational diabetes mellitus in late of pregnancy and 2 cases with preterm delivery. In total, 168 fetuses (85 male and 83 female) with 843 scanned observations were enrolled in this study. The mean GA at the first scan was 22.7 ± 1.3 weeks. Each fetus was scanned 3–6 times. The IAA was recorded in all observations, and the MAA was recorded in 752 observations, resulting in 100 and 89.2% acquisition rates for the IAA and MAA, respectively. The clinical characteristics of the cohort at recruitment and pregnancy outcomes are presented in Table 1.

**Table 1:** The clinical characteristics of the cohort and pregnancy outcomes (*n* = 168)

Parameters	Mean (range) or n (%)
Maternal:	
Age, years	27.5 (20–40)
BMI, kg/m^2^	23.2 (18–28.2)
Nulliparous, n	113 (67.3%)
Fetal:	
GA at first scan, weeks	22.7 (20–24)
EFW at first scan, g	520 (240–770)
GA at delivery, weeks	39.1 (37.1–41.1)
Birth weight, g	3197 (2639–3712)
Male:female	85:83
Outcome:	
Cesarean section, n	18 (10.7%)
Apgar score < 7 at 1 min	6 (3.5%)
Apgar score < 7 at 5 min	2 (1.2%)
NICU	7 (4.2%)
neonatal mortality	0

*BMI* Body-mass index, *DM* Diabetes mellitus, *NICU* Neonatal intensive care unit

**Fig. 2:**
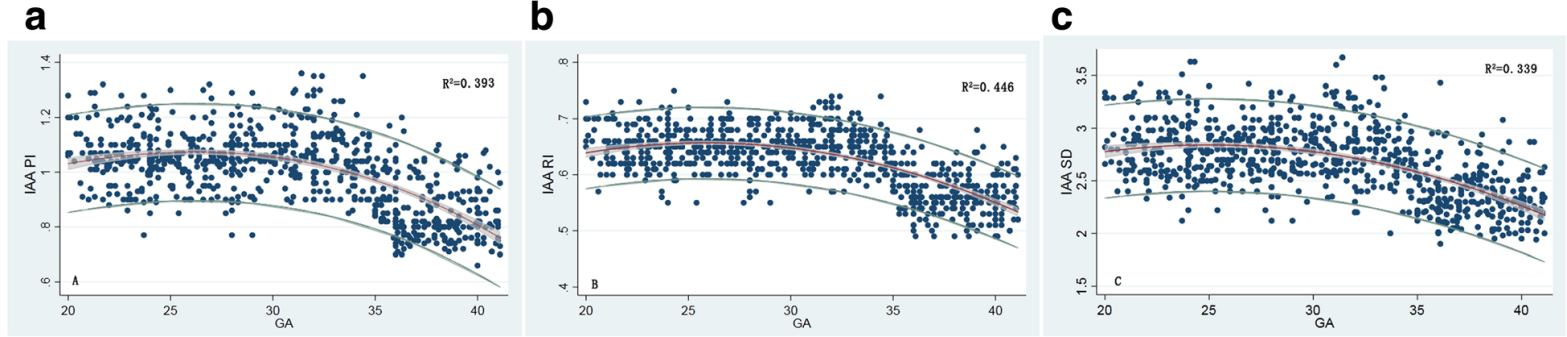
Smoothed 5th, 50th and 95th percentile curves of the **a**) pulsatility index, **b**) resistance index and **c**) systolic/diastolic ratio for the inferior adrenal artery. Blue circles show individual observations. Fitted percentiles according to gestational age in weeks are presented as solid lines. The 95% CI of the fitted mean value is presented as a gray area

The PI, RI and S/D of the IAA presented a second-degree fractional polynomial smoothing regression. Curve-fitted percentile charts for each Doppler index are shown in Fig. 2. GA-specific standard values for use in clinical practice for the 2.5th, 5th, 10th, 50th, 90th, 95th and 97.5th percentiles of the PI, RI, and S/D ratio for the IAA are presented in Tables 2, 3 and 4. The regression equations and the statistical formulas are shown in Supplementary file S1. There were no significant differences in Doppler indices between male and female fetuses at each gestational week. ANOVA demonstrated that the PI, RI and S/D of the IAA remained steady at 1.06 ± 0.1, 0.65 ± 0.03 and 2.80 ± 0.27, respectively, between 20 weeks and 34 weeks (*p* > 0.05), decreased significantly to 0.85 ± 0.1, 0.57 ± 0.04 and 2.35 ± 0.24 around 35 weeks (*p* < 0.001), and remained unchanged until term (*p* > 0.05).

**Table 2:** Longitudinal reference percentiles of pulsatility index (PI) for IAA

GA (weeks)	Percentile						
	2.5th	5th	10th	50th	90th	95th	97.5th
20	0.83	0.86	0.89	1.03	1.18	1.23	1.27
21	0.84	0.87	0.91	1.04	1.2	1.24	1.29
22	0.85	0.88	0.93	1.05	1.21	1.26	1.3
23	0.86	0.89	0.92	1.06	1.22	1.27	1.31
24	0.86	0.89	0.93	1.07	1.22	1.27	1.32
25	0.87	0.9	0.93	1.07	1.23	1.28	1.32
26	0.87	0.9	0.93	1.07	1.23	1.28	1.32
27	0.86	0.89	0.93	1.07	1.22	1.28	1.32
28	0.86	0.89	0.93	1.07	1.22	1.27	1.32
29	0.85	0.88	0.92	1.06	1.21	1.26	1.3
30	0.85	0.87	0.91	1.05	1.2	1.25	1.29
31	0.84	0.86	0.9	1.03	1.19	1.23	1.28
32	0.82	0.85	0.89	1.02	1.17	1.22	1.26
33	0.81	0.84	0.87	1	1.14	1.19	1.23
34	0.79	0.81	0.85	0.97	1.11	1.16	1.2
35	0.77	0.79	0.83	0.95	1.09	1.13	1.17
36	0.75	0.77	0.81	0.93	1.06	1.11	1.14
37	0.72	0.75	0.78	0.89	1.02	1.07	1.1
38	0.7	0.72	0.75	0.86	0.99	1.03	1.06
39	0.67	0.69	0.72	0.83	0.95	0.99	1.02
40	0.64	0.67	0.69	0.8	0.92	0.95	0.98
41	0.62	0.64	0.67	0.77	0.88	0.92	0.95

**Table 3:** Longitudinal reference percentiles of resistance index (RI) for IAA

GA (weeks)	Percentile						
	2.5th	5th	10th	50th	90th	95th	97.5th
20	0.57	0.58	0.59	0.64	0.69	0.7	0.72
21	0.57	0.58	0.6	0.65	0.7	0.71	0.72
22	0.57	0.59	0.6	0.65	0.7	0.71	0.73
23	0.58	0.59	0.6	0.65	0.7	0.72	0.73
24	0.58	0.59	0.6	0.66	0.71	0.72	0.73
25	0.58	0.59	0.61	0.66	0.7	0.72	0.73
26	0.58	0.59	0.61	0.66	0.71	0.72	0.73
27	0.58	0.59	0.61	0.66	0.71	0.72	0.73
28	0.58	0.59	0.6	0.65	0.7	0.72	0.73
29	0.57	0.59	0.6	0.65	0.7	0.71	0.73
30	0.57	0.58	0.6	0.65	0.7	0.71	0.72
31	0.57	0.58	0.59	0.64	0.69	0.7	0.72
32	0.56	0.57	0.58	0.64	0.68	0.7	0.71
33	0.55	0.56	0.58	0.63	0.68	0.69	0.7
34	0.54	0.55	0.57	0.62	0.67	0.68	0.69
35	0.53	0.55	0.56	0.61	0.66	0.67	0.68
36	0.52	0.54	0.55	0.6	0.65	0.66	0.68
37	0.51	0.52	0.54	0.59	0.64	0.65	0.66
38	0.5	0.51	0.52	0.57	0.62	0.64	0.65
39	0.48	0.5	0.51	0.56	0.61	0.63	0.64
40	0.47	0.48	0.5	0.55	0.6	0.61	0.62
41	0.46	0.47	0.48	0.53	0.59	0.6	0.61

**Table 4:** Longitudinal reference percentiles of systolic: diastolic ratio (S/D) for IAA

GA (weeks)	Percentile						
	2.5th	5th	10th	50th	90th	95th	97.5th
20	2.28	2.35	2.44	2.77	3.15	3.26	3.36
21	2.31	2.38	2.46	2.8	3.17	3.29	3.39
22	2.32	2.39	2.48	2.81	3.19	2.31	3.41
23	2.33	2.4	2.49	2.83	3.21	3.32	3.43
24	2.33	2.41	2.5	2.83	3.21	3.33	3.44
25	2.33	2.41	2.5	2.83	3.21	3.33	3.44
26	2.33	2.4	2.49	2.83	3.21	3.33	3.43
27	2.32	2.4	2.48	2.82	3.2	3.31	3.42
28	2.31	2.38	2.47	2.8	3.18	3.3	3.4
29	2.29	2.36	2.45	2.78	3.15	3.27	3.37
30	2.27	2.34	2.42	2.75	3.12	3.23	3.34
31	2.24	2.31	2.4	2.72	3.09	3.2	3.3
32	2.21	2.28	2.37	2.69	3.05	3.16	3.26
33	2.17	2.24	2.33	2.64	3	3.1	3.2
34	2.13	2.2	2.28	2.59	2.94	3.05	3.15
35	2.09	2.16	2.24	2.54	2.89	2.99	3.09
36	2.05	2.12	2.2	2.49	2.83	2.94	3.03
37	2	2.06	2.14	2.43	2.76	2.86	2.95
38	1.94	2	2.08	2.37	2.69	2.79	2.88
39	1.89	1.95	2.02	2.31	2.63	2.72	2.81
40	1.83	1.89	1.97	2.24	2.55	2.65	2.74
41	1.79	1.84	1.91	2.19	2.5	2.59	2.68

**Fig. 3:**
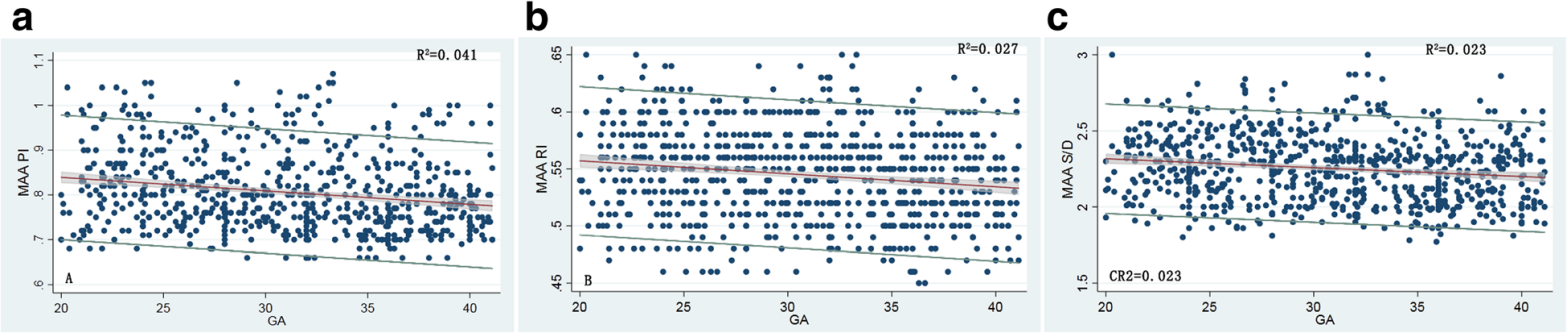
Smoothed 5th, 50th and 95th percentile curves of the **a**) the pulsatility index, **b**) the resistance index and **c**) the systolic/diastolic ratio for the middle adrenal artery. Blue circles show individual observations. Fitted percentiles according to gestational age in weeks are presented as solid lines. The 95% CI of the fitted mean value is presented as a gray area

The PI, RI and S/D of the MAA, in summary, remained unchanged at 0.81 ± 0.09, 0.55 ± 0.04 and 2.25 ± 0.22, respectively, during the second half of pregnancy, based on the scatter graph (Fig. 3) and very small R^2^ in the regression analysis (R^2^ = 0.041, 0.027 and 0.023, respectively). No difference in each Doppler index was found between male and female fetuses. The regression equations and the statistical formulas are shown in Supplementary file S2. GA-specific references for the 2.5th, 5th, 10th, 50th, 90th, 95th and 97.5th percentiles of the PI, RI, and S/D ratio for the MAA are presented in Supplementary file S3–1, S3–2 and S3–3 separately.

**Fig. 4:**
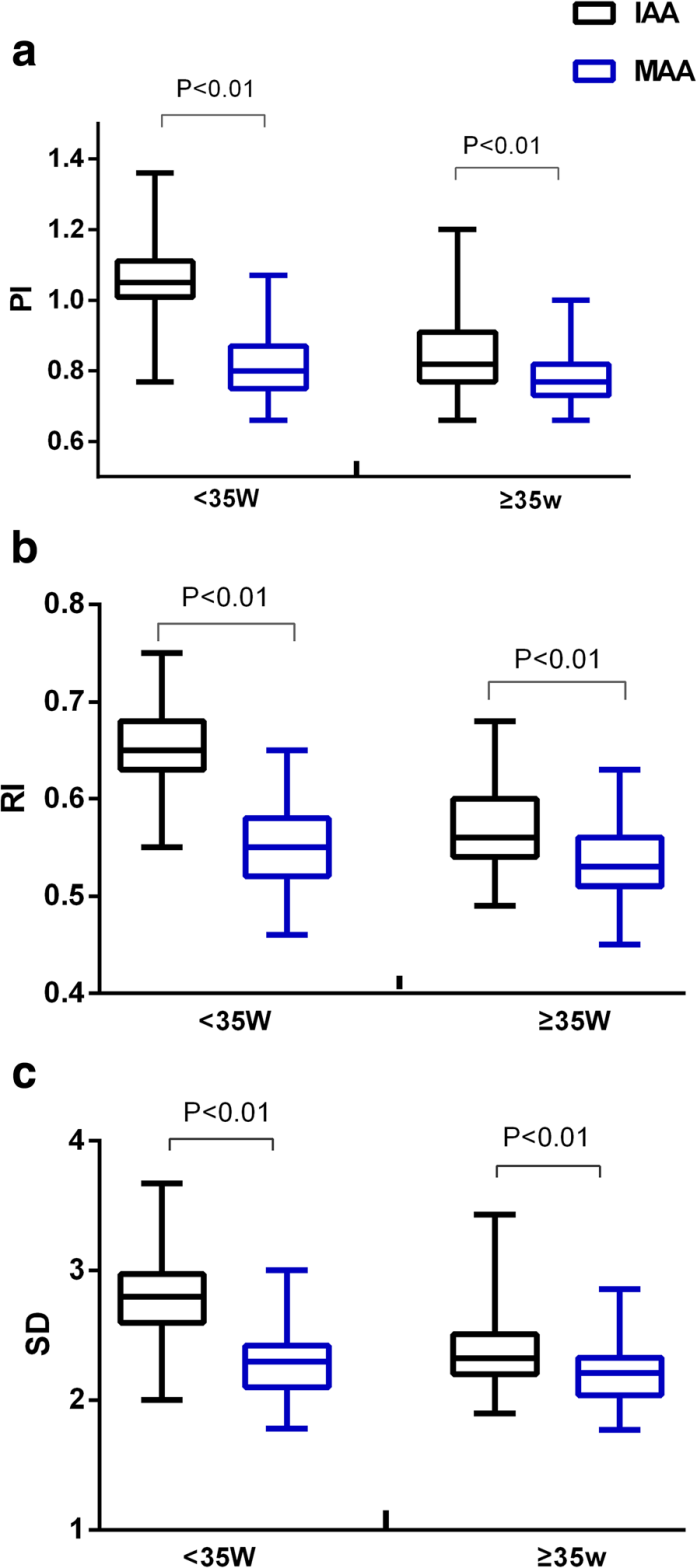
**a**) Pulsatility index (PI); **b**) resistance index and **c**) systolic/diastolic ratio from paired observations of the IAA and MAA. The PI RI and S/D of the MAA were significantly lower than those of the IAA, even after 35 weeks of gestation. IAA, inferior adrenal artery; MAA, middle adrenal artery

Comparing the paired 752 observations between the IAA and MAA, the PI RI and S/D of the MAA were significantly lower than those of the IAA, even after 35 weeks of gestation (Fig. 4).

The ICCs for the PI, RI, and S/D of the IAA were 91.4% (95% CI, 86.9–94.4%), 88% (95% CI, 81.9–90.1%), and 92.3% (95% CI, 88.2–95%), respectively. The ICCs for the PI, RI, and S/D of the MAA were 93.4% (95% CI, 89.7–95.8%), 89.2% (95% CI, 83.2–93.1%), and 90.3% (95% CI, 85.2–93.6%), respectively.

## Discussion

This study showed the following. First, we established GA-specific reference ranges of adrenal artery Doppler indices that were based on longitudinal observations. Second, there is a zonal difference in blood supply in favor of the FZ based on the observation of lower resistance in the MAA than in the IAA.

Furthermore, according to Royston P’s theory [11], longitudinal studies of fetal growth require half the sample size of a cross-sectional study to estimate a given percentile with the same precision. Hence, our cohort of fetuses, which is the largest cohort to date and contributed 843 and 752 measures of the IAA and MAA, has power equivalent to a sample of at least 1500 measures in a cross-sectional study.

To our knowledge, this is the first report about the Doppler characteristics of the IAA in the fetal adrenal gland. In summary, the PI, RI and S/D of the IAA remained constant between 20 weeks and 34 weeks, decreased significantly at 35 weeks and maintained this low resistance until term. This feature indicates that there is more blood flowing to the outer zone of the gland after 35 gestational weeks. This increased blood flow to this part of the gland in the last 6 weeks of pregnancy may contribute to the functional development of the neocortex. As mentioned above, the neocortex comprises the TZ and DZ, which are believed to differentiate into the cortical and fasciculate zones of the postnatal adrenal, respectively. The neocortex has been considered to become steroidogenically active in the late phase of pregnancy. However, de novo cortisol production likely occurs transiently early in pregnancy (approximately 7–10 weeks of gestation) and appears to be suppressed until late gestation due to the lack of HSD3B2 expression [1]. Murphy BE [12] measured human fetal cortisol levels in umbilical cord serum under various modes of delivery at various GAs. They observed a drop from 8.4 ng/ml at 15–17 weeks to 4 ng/ml at 17^1/2^–20 weeks, followed by a steep rise to 20 ng/ml by 35–36 weeks and a further increase to 45.1 ng/ml between 37^1/2^ and 40 weeks. This midgestational fall and steep late gestational rising pattern of fetal serum cortisol levels were independent of sex and delivery mode. On the other hand, aldosterone synthesis in the fetal adrenal gland may be suppressed during mid-gestation due to the lack of HSD3B and CYP11B2 transcripts [2, 13] but likely becomes active by late gestation [14]. Johnston ZC [13] observed no detectable aldosterone from 109 human intra-adrenal samples ranging from 11 to 21 gestational weeks. Martinerie L’s study [14] demonstrated that preterm infants (26–36 weeks) had obviously lower levels of plasma and urinary aldosterone than infants born ≥37 weeks. Together, these findings indicate that the capacity of the fetal adrenal to generate significant amounts of aldosterone also begins in the last few weeks of pregnancy. Therefore, the decreased resistance and subsequent increased blood flow of the IAA in late gestation may contribute to the surge in steroidogenic activity in the DZ and TZ during the corresponding period, facilitating the delivery of tropic agents, steroid hormone precursors and hormone products.

Our present study demonstrated that the PI, RI and S/D of the MAA were lower than those of the IAA. Moreover, the low resistance of the MAA remained stable during the second half of pregnancy. The zonal difference in blood redistribution in favor of the FZ relates to the structural enlargement and steroidogenic activity of the FZ during the fetal period. First, the human adrenal gland continues to grow rapidly in utero, and its weight increases almost 10-fold during the first trimester [15], with a size as large as the fetal kidney by 20 weeks gestation, a size 10- to 20-fold larger that of the adult adrenal gland by 30 gestational weeks and a weight of approximately 3–5 g by full term [16]. Rapid adrenal cortex growth is almost always due to enlargement of the FZ [1], while the outer DZ remains relatively constant. Second, the steroidogenic activity of the outer zone surges by late gestation, while the inner FZ appears to be the most steroidogenically active zone throughout most of gestation [17]. The FZ, which expresses CYP11A and CYP17 but not HSD3B2, is responsible for the production of mainly adrenal androgens, especially dehydroepiandrosterone (DHEA) and DHEA sulfate (DHEAS). DHEAS production begins at approximately 8–10 weeks of gestation, continues to increase considerably during the second and third trimesters, and is up to approximately 200 mg per day by term [1]. DHEAS acts as an essential precursor for placental conversion to estrogens, which is crucial for intrauterine homeostasis, fetal maturation and labor activation.

The MAA was detected in 89.2% of observations (752 out of 843 observations). Its congenital absence [18] and nearly vertical course [10] from the aorta are the main contributors. In the present study, the Doppler indices of the MAA showed little change as a function of GA (r^2^ = 0.04, 0.02 and 0.02 for PI, RI and S/D, respectively). As mentioned above, the FZ kept enlargement and hormone secretory activity throughout most of gestation. Therefore, such constant low resistance of MAA during the whole fetal period is beneficial for providing sufficient bool blow to FZ in utero. Moreover, the initial very low resistance in MAA may limit the ability for further reduction in late of pregnancy. However, this was inconsistent with previous observations; Mari G [6] reported that MAA-PI decreased linearly with advancing GA (*r* = 0.46), and Fujita Y [9] reported that MAA-RI increased until 31 weeks and decreased thereafter. This discrepancy may be associated with the type, methodology, and sample size of each survey. Mari G’s study [6] was based on cross-sectional data. Fujita Y’s study [9] had common methodologic problems, such as insufficient information about design and a failure to account for within-subject changes.

This work has two limitations. First, we performed Doppler measurements only on the unilateral adrenal gland, which is close to the probe. Thus, we were unable to observe the difference between the left and right glands; however, this examination takes no more than 5 min for each fetus and minimizes fetal insonation. Second, we demonstrated no sex difference in Doppler indices of the fetal adrenal gland during the second half of pregnancy, consistent with the absence of major differences in the expression of hormone concentrations [12, 13] or steroidogenic enzymes [19] between male and female fetuses. However, whether there is a sex difference in early pregnancy before sexual differentiation remains unclear. More surveys on the first and early second trimesters will be needed.

## Conclusion

GA-specific reference ranges of adrenal artery Doppler indices (PI, RI, and S/D) were established based on longitudinal observations. There is a zonal difference in blood supply in favor of the FZ. The characteristics of Doppler indices in relation to GA and adrenal zone may indicate the development of steroidogenic activity in the fetal adrenal gland. Such observations could be more appropriate for serial evaluation of fetal hemodynamics.

## Supplementary Information

**Additional file 1 S1**: Fitting equations and parameters of computations for percentiles for IAA Doppler indices according to gestational age (GA) in weeks. **S3-1**: Longitudinal reference percentiles of pulsatility index (PI) for MAA. **S3-2**: Longitudinal reference percentiles of resistance index (RI) for MAA. **S3-3**: Longitudinal reference percentiles of systolic: diastolic ratio (S/D) for MAA.

## Data Availability

The datasets used and/or analyzed during the current study are available from the corresponding author on reasonable request.

## Abbreviations

PI: Pulsatility index
RI: Resistance index
S/D: Systolic:diastolic ratio
IAA: Inferior adrenal artery
MAA: Middle adrenal artery
FZ: Fetal zone
DZ: Definitive zone
