# Supplementary material for: Zone-specific reference ranges of fetal adrenal artery Doppler indices: a longitudinal study
Source: BMC Pregnancy Childbirth. 2020 Dec 11;20:774. doi: 10.1186/s12884-020-03480-z (PMC7733276; doi:10.1186/s12884-020-03480-z)
Supplement: Supplementary file 1 — Additional file 1 S1: Fitting equations and parameters of computations for percentiles for IAA Doppler indices according to gestational age (GA) in weeks. S3-1: Longitudinal reference percentiles of pulsatility index (PI) for MAA. S3-2: Longitudinal reference percentiles of resistance index (RI) for MAA. S3-3: Longitudinal reference percentiles of systolic: diastolic ratio (S/D) for MAA. [file 12884_2020_3480_MOESM1_ESM.docx]

S1:Fitting equations and parameters of computations for percentiles for IAA Doppler indices according to gestational age (GA) in weeks

| Variables | equation or parameters |
| --- | --- |
| pulsatility index (PI) | Fitting equation X=0.6575949+0.0045648*GA^2^-0.0012121*ln(GA)*GA^2^ (R^2^=0.393, P<0.05) |
|  | Multilevel modeling λ=0 |
|  | µ_z_=-1.075448+1.065567*X |
|  | σ_z_^2^=4.18e^-14^+5.44e^-17^*X^2^+0.0117327 |
|  | GA-specific reference value with 95% coverage exp(µ_z_±1.645σ_z_) |
| resistance index(RI) | Fitting equation X=0.3043318+0.0273159*GA-0.000529*GA^2^  ( R^2^=0.446, P<0.05) |
|  | Multilevel modeling λ=1 |
|  | µ_z_=-0.0011839+1.001848*X |
|  | σ_z_^2^=0.0050461+0.0085368*X^2^-0.0110272*X |
|  | GA-specific reference value with 95% coverage µ_z_±1.645σ_z_ |
| systolic: diastolic ratio (S/D) | Fitting equation X=1.233899+0.128664*GA-0.0025741*GA^2^ (R^2^=0.339, P<0.05) |
|  | Multilevel modeling λ=0 |
|  | µ_z_=-0.0671342+0.3900993*X |
|  | σ_z_^2^＝0.0283998+0.0023926*X^2^-0.0133704*X |
|  | GA-specific reference value with 95% coverage exp(µ_z_±1.645σ_z_) |

S2: Fitting equations and parameters of computations for percentiles for MAA Doppler indices according to gestational age (GA) in weeks

| Variables | equation or parameters |
| --- | --- |
| pulsatility index (PI) | Fitting equation X=0.8995171-0.0030155*GA (R^2^=0.041 P<0.05) |
|  | Multilevel modeling λ=-2.378541 |
|  | µ_z_=5.64974-4.848657*X |
|  | σ_z_^2^=0.1550325+0.0000284*X^2^-0.000047*X |
|  | GA-specific reference value with 95% coverage (µ_z_±1.645σ_z_)^1/λ^ |
| resistance index(RI) | Fitting equation X=0.5799491-0.0011405*GA (R^2^=0.027 P<0.05) |
|  | Multilevel modeling λ=0 |
|  | µ_z_=-1.597333+1.812276*X |
|  | σ_z_^2^= 0.0051905+0.0002649*X^2^ |
|  | GA-specific reference value with 95% coverage exp(µ_z_±1.645σ_z_) |
| systolic: diastolic ratio (S/D) | Fitting equation X=2.434645-0.0058661*GA (R^2^=0.023 P<0.05) |
|  | Multilevel modeling λ=0 |
|  | µ_z_=-0.1871846+0.4414975*X |
|  | σ_z_^2^＝0.0092054+0.0000164*X^2^ |
|  | GA-specific reference value with 95% coverage exp(µ_z_±1.645σ_z_) |

S3-1:Longitudinal reference percentiles of pulsatility index (PI) for MAA

| **GA**  **(weeks)** | **Percentile**  **2.5^th^ 5^th^ 10^th^ 50^th^ 90^th^ 95^th^ 97.5th** | | | | | | |
| --- | --- | --- | --- | --- | --- | --- | --- |
| 20 | 0.7 | 0.71 | 0.73 | 0.82 | 0.97 | 1.03 | 1.09 |
| 21 | 0.7 | 0.71 | 0.73 | 0.82 | 0.96 | 1.02 | 1.08 |
| 22 | 0.69 | 0.71 | 0.73 | 0.82 | 0.96 | 1.01 | 1.07 |
| 23 | 0.69 | 0.71 | 0.73 | 0.81 | 0.95 | 1 | 1.07 |
| 24 | 0.69 | 0.71 | 0.73 | 0.81 | 0.95 | 1 | 1.06 |
| 25 | 0.69 | 0.7 | 0.72 | 0.81 | 0.94 | 1 | 1.05 |
| 26 | 0.69 | 0.7 | 0.72 | 0.81 | 0.94 | 0.99 | 1.04 |
| 27 | 0.68 | 0.7 | 0.72 | 0.8 | 0.93 | 0.98 | 1.04 |
| 28 | 0.68 | 0.7 | 0.72 | 0.8 | 0.93 | 0.98 | 1.03 |
| 29 | 0.68 | 0.7 | 0.71 | 0.8 | 0.92 | 0.97 | 1.02 |
| 30 | 0.68 | 0.69 | 0.71 | 0.79 | 0.92 | 0.97 | 1.02 |
| 31 | 0.68 | 0.69 | 0.71 | 0.79 | 0.91 | 0.96 | 1.01 |
| 32 | 0.68 | 0.69 | 0.71 | 0.79 | 0.91 | 0.96 | 1 |
| 33 | 0.67 | 0.69 | 0.71 | 0.79 | 0.9 | 0.95 | 1 |
| 34 | 0.67 | 0.69 | 0.7 | 0.78 | 0.9 | 0.95 | 0.99 |
| 35 | 0.67 | 0.69 | 0.7 | 0.78 | 0.9 | 0.94 | 0.99 |
| 36 | 0.67 | 0.68 | 0.7 | 0.78 | 0.89 | 0.94 | 0.98 |
| 37 | 0.67 | 0.68 | 0.7 | 0.77 | 0.89 | 0.93 | 0.97 |
| 38 | 0.67 | 0.68 | 0.7 | 0.77 | 0.88 | 0.93 | 0.97 |
| 39 | 0.67 | 0.68 | 0.7 | 0.77 | 0.88 | 0.92 | 0.96 |
| 40 | 0.66 | 0.68 | 0.69 | 0.77 | 0.88 | 0.92 | 0.96 |
| 41 | 0.66 | 0.68 | 0.69 | 0.77 | 0.87 | 0.91 | 0.95 |

S3-2:Longitudinal reference percentiles of resistance index (RI) for MAA

| **GA**  **(weeks)** | **Percentile**  **2.5^th^ 5^th^ 10^th^ 50^th^ 90^th^ 95^th^ 97.5th** | | | | | | |
| --- | --- | --- | --- | --- | --- | --- | --- |
| 20 | 0.48 | 0.49 | 0.51 | 0.55 | 0.61 | 0.63 | 0.64 |
| 21 | 0.48 | 0.49 | 0.5 | 0.55 | 0.61 | 0.62 | 0.64 |
| 22 | 0.48 | 0.49 | 0.5 | 0.55 | 0.61 | 0.62 | 0.64 |
| 23 | 0.48 | 0.49 | 0.5 | 0.55 | 0.61 | 0.62 | 0.64 |
| 24 | 0.48 | 0.49 | 0.5 | 0.55 | 0.6 | 0.62 | 0.64 |
| 25 | 0.48 | 0.49 | 0.5 | 0.55 | 0.6 | 0.62 | 0.63 |
| 26 | 0.48 | 0.49 | 0.5 | 0.55 | 0.6 | 0.62 | 0.63 |
| 27 | 0.47 | 0.49 | 0.5 | 0.55 | 0.6 | 0.62 | 0.63 |
| 28 | 0.47 | 0.48 | 0.5 | 0.55 | 0.6 | 0.62 | 0.63 |
| 29 | 0.47 | 0.48 | 0.5 | 0.55 | 0.6 | 0.61 | 0.63 |
| 30 | 0.47 | 0.48 | 0.5 | 0.54 | 0.6 | 0.61 | 0.63 |
| 31 | 0.47 | 0.48 | 0.49 | 0.54 | 0.6 | 0.61 | 0.63 |
| 32 | 0.47 | 0.48 | 0.49 | 0.54 | 0.6 | 0.61 | 0.62 |
| 33 | 0.47 | 0.48 | 0.49 | 0.54 | 0.59 | 0.61 | 0.62 |
| 34 | 0.47 | 0.48 | 0.49 | 0.54 | 0.59 | 0.61 | 0.62 |
| 35 | 0.47 | 0.48 | 0.49 | 0.54 | 0.59 | 0.6 | 0.62 |
| 36 | 0.47 | 0.48 | 0.49 | 0.54 | 0.59 | 0.61 | 0.62 |
| 37 | 0.46 | 0.48 | 0.49 | 0.54 | 0.59 | 0.6 | 0.62 |
| 38 | 0.46 | 0.47 | 0.49 | 0.53 | 0.59 | 0.6 | 0.62 |
| 39 | 0.46 | 0.47 | 0.49 | 0.53 | 0.59 | 0.6 | 0.62 |
| 40 | 0.46 | 0.47 | 0.49 | 0.53 | 0.58 | 0.6 | 0.61 |
| 41 | 0.46 | 0.47 | 0.48 | 0.53 | 0.58 | 0.6 | 0.61 |

S3-3:Longitudinal reference percentiles of systolic: diastolic ratio (S/D) for MAA

| **GA**  **(weeks)** | **Percentile**  **2.5^th^ 5^th^ 10^th^ 50^th^ 90^th^ 95^th^ 97.5th** | | | | | | |
| --- | --- | --- | --- | --- | --- | --- | --- |
| 20 | 1.91 | 1.97 | 2.04 | 2.3 | 2.61 | 2.7 | 2.78 |
| 21 | 1.9 | 1.96 | 2.03 | 2.3 | 2.6 | 2.7 | 2.78 |
| 22 | 1.9 | 1.96 | 2.03 | 2.3 | 2.6 | 2.7 | 2.77 |
| 23 | 1.9 | 1.95 | 2.02 | 2.29 | 2.59 | 2.68 | 2.76 |
| 24 | 1.89 | 1.95 | 2.02 | 2.28 | 2.58 | 2.67 | 2.76 |
| 25 | 1.88 | 1.94 | 2.01 | 2.27 | 2.57 | 2.67 | 2.75 |
| 26 | 1.88 | 1.94 | 2.01 | 2.27 | 2.57 | 2.66 | 2.74 |
| 27 | 1.87 | 1.93 | 2 | 2.26 | 2.56 | 2.65 | 2.73 |
| 28 | 1.87 | 1.93 | 2 | 2.26 | 2.56 | 2.65 | 2.73 |
| 29 | 1.86 | 1.92 | 1.99 | 2.25 | 2.55 | 2.64 | 2.72 |
| 30 | 1.86 | 1.92 | 1.98 | 2.25 | 2.54 | 2.63 | 2.71 |
| 31 | 1.85 | 1.91 | 1.98 | 2.24 | 2.54 | 2.63 | 2.71 |
| 32 | 1.84 | 1.9 | 1.97 | 2.23 | 2.52 | 2.61 | 2.69 |
| 33 | 1.84 | 1.9 | 1.96 | 2.22 | 2.51 | 2.6 | 2.68 |
| 34 | 1.84 | 1.9 | 1.96 | 2.22 | 2.51 | 2.6 | 2.68 |
| 35 | 1.84 | 1.9 | 1.96 | 2.22 | 2.51 | 2.6 | 2.68 |
| 36 | 1.83 | 1.89 | 1.95 | 2.22 | 2.5 | 2.59 | 2.67 |
| 37 | 1.83 | 1.88 | 1.95 | 2.21 | 2.5 | 2.58 | 2.66 |
| 38 | 1.82 | 1.88 | 1.94 | 2.2 | 2.49 | 2.58 | 2.66 |
| 39 | 1.82 | 1.87 | 1.94 | 2.19 | 2.48 | 2.57 | 2.65 |
| 40 | 1.81 | 1.87 | 1.93 | 2.19 | 2.48 | 2.56 | 2.64 |
| 41 | 1.81 | 1.86 | 1.93 | 2.18 | 2.47 | 2.56 | 2.64 |
